# Case report: A rare combination of aldosterone-secreting adrenocortical carcinoma and papillary thyroid carcinoma with Graves’ disease

**DOI:** 10.3389/fendo.2024.1310408

**Published:** 2024-04-05

**Authors:** Yuhai Zhang, Jingwen Yu, Cunxia Fan, Fei Wang, Haiwei Liu, Kaining Chen

**Affiliations:** Department of Endocrinology, Hainan General Hospital, Hainan Affiliated Hospital of Hainan Medical University, Haikou, Hainan, China

**Keywords:** adrenocortical carcinoma, aldosterone, exome sequencing, Graves’ disease, papillary thyroid carcinoma

## Abstract

Adrenocortical carcinoma (ACC) is a rare malignancy originating in the adrenal glands, aldosterone-producing ACC, even rarer. Papillary thyroid carcinoma (PTC), by contrast, accounts for the majority of thyroid carcinomas. We herein describe the first reported case of a female with comorbidities of aldosterone-producing ACC, PTC, and Graves’ Disease(GD). The patient achieved transient clinical remission following adrenalectomy. However, three months later, aldosterone-producing ACC lung metastases emerged. Subsequently, within another three-month interval, she developed thyroid eye disease(TED). The patient died roughly one year after the adrenal operation. Exome sequencing did not reveal associations between aldosterone-producing ACC, PTC, and GD, and the underlying concurrence mechanism has yet to be elucidated. Further research of similar cases are needed to confirm potential links between the three pathologies.

## Introduction

Adrenocortical carcinoma (ACC) is a rare endocrine malignancy with a population incidence ranging from 0.001 to 0.002% (1). Aldosterone-producing ACC, a subtype of ACC, is rarer. Papillary thyroid carcinoma (PTC), a slowly progressive cancer with a high survival rate, represents more than 70–90% of thyroid malignancies (2–4). Graves’ disease (GD) is an autoimmune system disorder that results in the overproduction of thyroid hormone When two or more endocrine malignancies are detected in the same person, genetic disorders should be ruled out. However, there is limited insight detailing the association between ACC and PTC (5). Moreover, there are no published reports on the association between ACC and PTC with GD.

Here, we report a unique case of a patient with aldosterone-producing ACC who, surprisingly, also had a localized PTC and GD. The primary concern was the simultaneous appearance of two endocrine tumors, and the possibility of hereditary cancer syndrome.

## Methods and results

### Case presentation

A 45-year-old female was referred to our department with complaints of fatigue and high blood pressure(158/90mmHg). Lab test showed refractory hypokalemia with a minimum level of 1.97mmol/L(normal range: 3.5-5.3mmol/L). Anti-hypertensive drug and potassium supplements were prescribed to her but of limited effectiveness. There were no features suggestive of Cushing’s syndrome (CS) or pheochromocytoma on this patient. She was admitted with a family history of hypertension. Physical examination revealed 135/80 mmHg blood pressure, tremor, grade II thyroid enlargement, and multiple thyroid nodules. A mass was palpable in the upper left quadrant of the abdomen.

**Figure 1:**
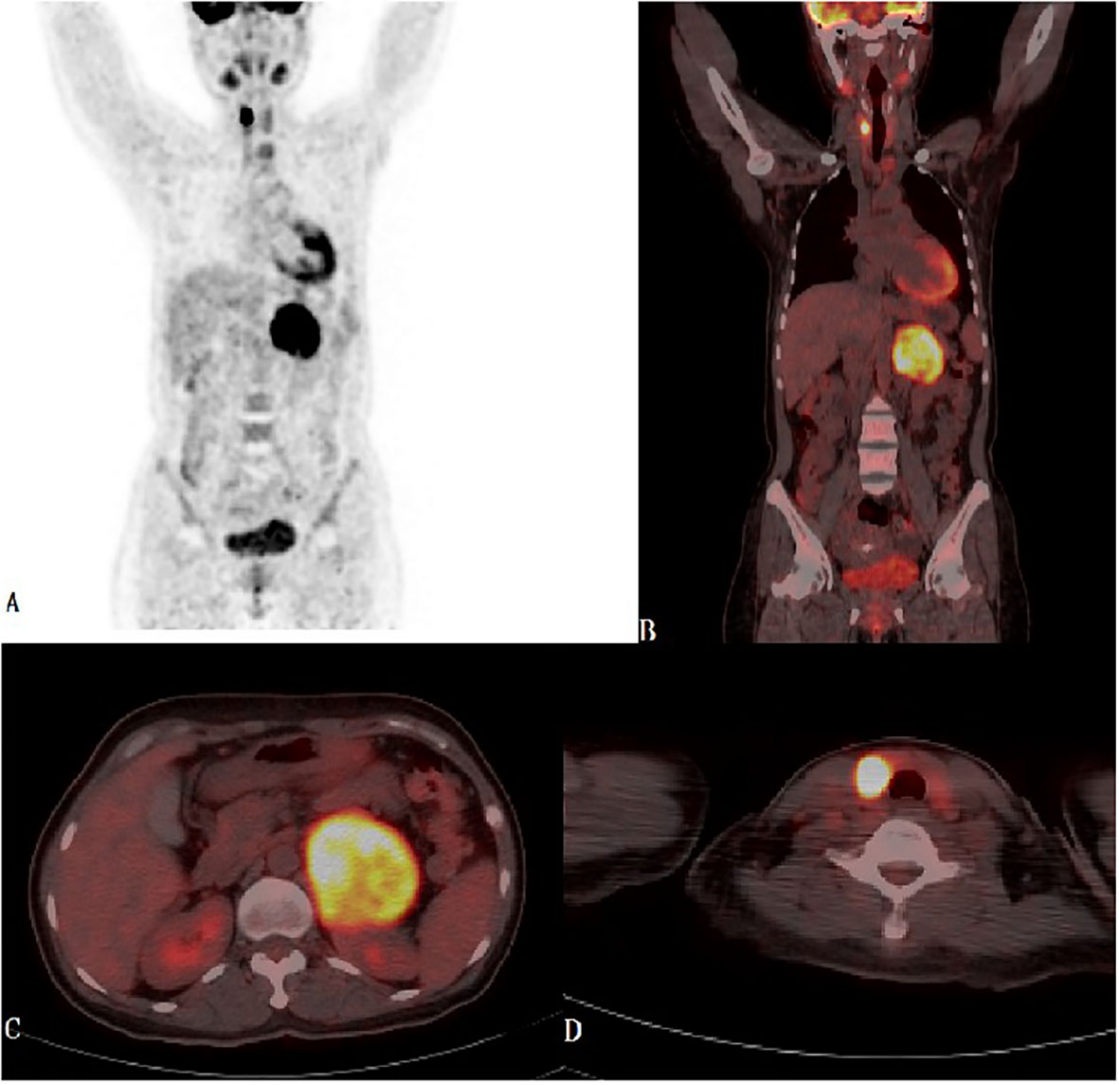
**(A-D)** Maximum Intensity Projection-MIP-PET and PET-CT fused images in coronal and transaxial projections showed left bulky retroperitoneal mass with heterogeneous intense FDG uptake and focal intense FDG uptake in the right thyroid lobe.

Laboratory tests demonstrated consistent hypokalemia and an elevated aldosterone-renin ratio (ARR = 3.2, see **Table 1**). The captopril challenge test did not inhibit aldosterone levels (**Table 2**), and the adrenocorticotropic hormone-cortisol rhythm was normal. Thyroid function test revealed elevated FT3 and FT4, decreased TSH and positive TRAb (**Table 1**). Sex steroid, HbA1c and metanephrine levels were negative. Thyroid ultrasound showed abundant blood flow, three solid hypoechoic nodules(maximum size: 11×12 ×17 mm) and two predominantly cystic nodules in the right lobe(maximum size: 5.9×4.9mm), left lobe and isthmus were negative for nodules. Contrast-enhanced computed tomography (CECT) of the upper abdomen revealed a mass with clear margins in the left adrenal area, 67 mm x 70 mm in size (CT value: 37 HU, on plain scan). Fluorodeoxyglucose (FDG)-positron emission tomography/CT revealed intense FDG uptake in the left adrenal and right lobes of the thyroid gland (**Figure 1**). The fine needle aspiration biopsy(FNAB) of the biggest solid thyroid nodule was papillary thyroid carcinoma and was positive for BRAF-V600E mutation. Diagnoses were made as follows: 1. Adrenal cortical carcinoma? 2. Papillary thyroid carcinoma 3. Graves’ disease. Mineralocorticoid receptor antagonists(MRAs) and methimazole(MMZ) were given to the patient for normalization of serum potassium and thyroid function before surgery.

**Table 1:** Biochemical evaluation of the patient.

Parameters	Result	Normal range
Serum K^+^ (mmol/L)	2.38	3.5-5.3mmol/L
Serum Na^+^ (mmol/L)	140.5	137-147mmol/L
PAC (ng/dL)	52	7-30ng/dL
PRC(mU/L)	16.2	4.2-45.6mU/L
PAC/PRC	3.2	<2.0
24 hours urine K^+^ (mmol/h)	98.54	25-100mmol/h
FT3 (pmol/L)	6.2	2.43-6.01pmol/L
FT4 (pmol/L)	21.66	9.01-19.05pmol/L
TSH (mIU/L)	0.0027	0.35-4.94mIU/L
TRAb (IU/L)	32.25	≤1.75IU/L
Metanephrine (nmol/L)	0.32	≤0.50nmol/L
Normetanephrine (nmol/L)	0.54	≤0.90nmol/L
3-Methoxy tyramine (nmol/L)	<0.08	<0.18nmol/L
24 hours free urinary cortisol (ug)	20.6	3.5-45.0ug/24h

PAC, plasma aldosterone concentration; PRC, plasma renin concentration; FT3, free triiodothyronine; FT4, free thyroxine; TSH, thyroid-stimulating hormone; TRAb, thyrotropin receptor antibody.

**Table 2:** Results of Captopril-challenge Test.

	Before Captopril	1hr after Captopril	2hrs after Captopril
Aldosterone(ng/dl)	703	744	738
Cortisol(nmol/L)	203	205	181

**Figure 2:**
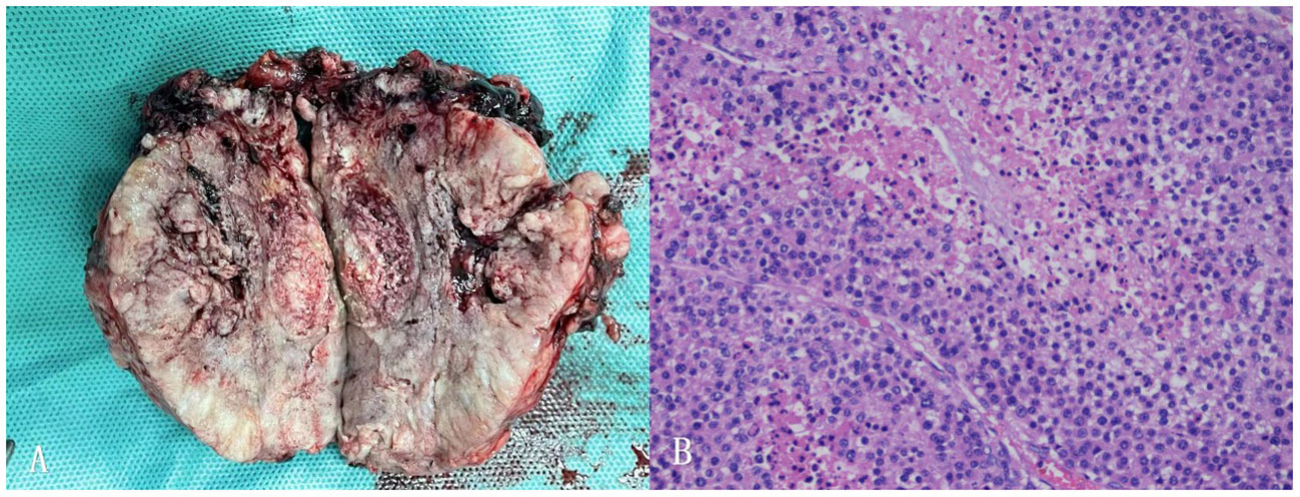
**(A)** Postoperative picture of the left adrenal mass specimen. **(B)** Histopathology of adrenocortical carcinoma of this patient (40x).

Adrenalectomy was performed using the Da Vinci Surgical System after the patients reached normal level of FT3 and FT4. The left adrenal gland, mostly occupied by a well-encapsulated tumor of 95 × 80 × 47 mm with no surrounding invasion, was completely resected. No liver surface metastases or direct invasion were observed. The periadrenal adipose tissue and lymph nodes were excised to ensure surgical clearance of R0. The gross specimen showed a coated surface, grayish-white and brown in color with hemorrhage, calcification, and cyst vacuolation. Histopathological examination revealed ACC. Immunohistochemical tests confirmed ACC with 90% Ki67 in the resected tissue (**Figure 2**).

**Figure 3:**
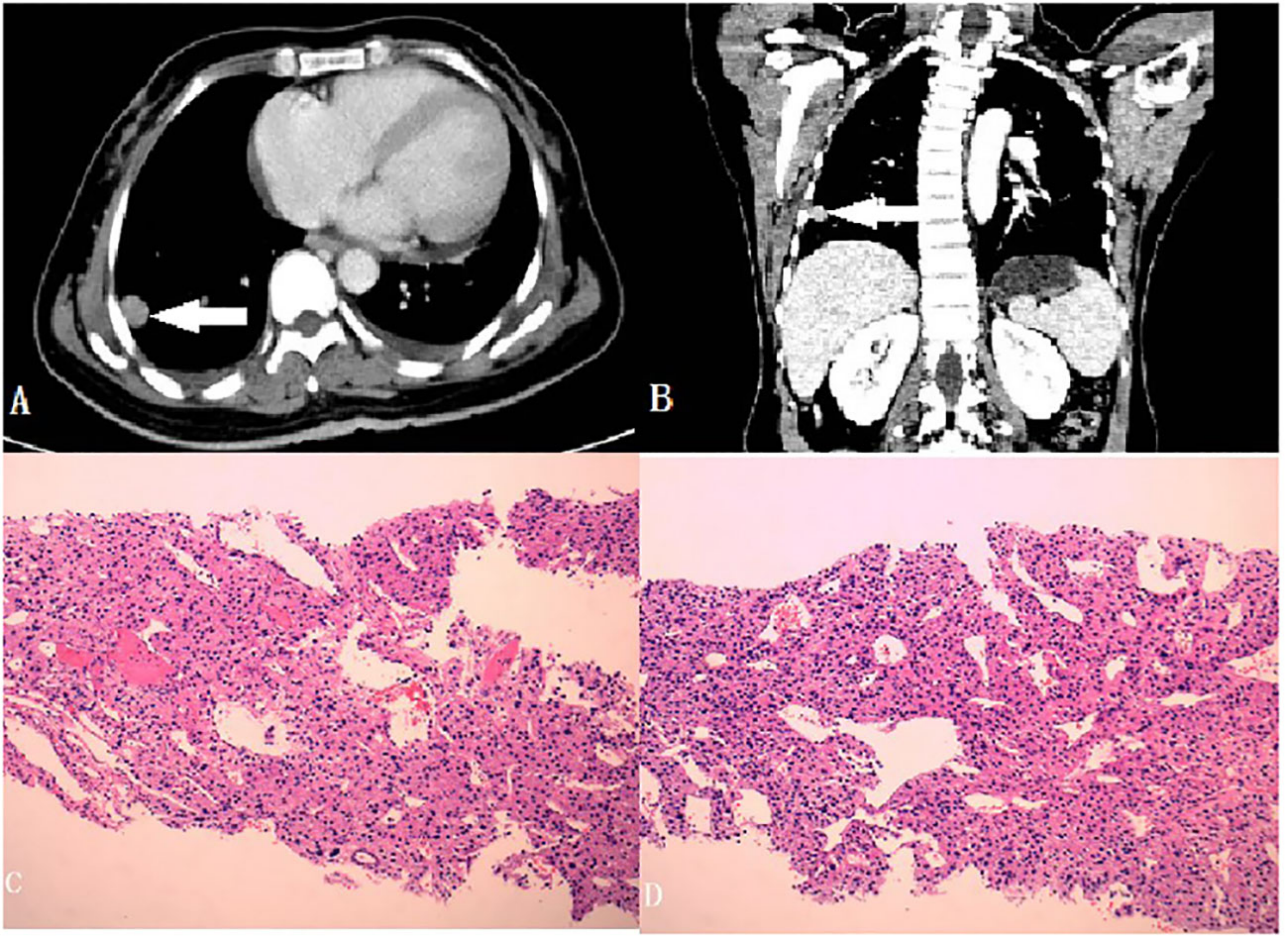
**(A, B)** Metastasizing nodules of the lung (Cross and Coronal section, with a white arrow to locate the foci); **(C, D)** Histopathology of the nodules (40x).

Blood pressure and potassium levels returned to normal postoperatively. Mitotane, prescribed to prevent tumor recurrence. The patient experienced severe nausea and vomiting administering mitotane. She found it very difficult to eat anything including the anti-hyperthyroidism tablets. Moreover, as she was not feeling any obvious discomfort stemming from her hyperthyroidism, she chose to discontinue the medication for her hyperthyroidism, as well as mitotane soon afterwards. Adjuvant chemotherapy was not administered. Three months later, a lung CT indicated multiple metastases and biopsy confirmed ACC metastasis, with Ki67 detectable in approximately 40% of the tissue (**Figure 3**). The patient developed severe hypokalemia again simultaneously. As her condition weakened progressively due to the deterioration of the ACC, the initially planned PTC surgery was cancelled, as well as chemotherapy for the metastases. Three months later she was also symptomatic of thyroid eye disease due to withdrawal of anti-hyperthyroidism drugs. They patient finally died of severe electrolyte disturbance and complications of the cancer one year after her first surgery.

### Whole exome sequencing

Genetic testing in blood and tissue samples was conducted to exclude possible hereditary targets contributing to the combination of ACC and PTC, specifically Li-Fraumeni syndrome (LFS) and multiple endocrine neoplasia 1 (MEN-1). However, gene mutation analysis revealed a heterozygous mutation of TP53 (c.1051A>G) and MEN-1 (c.1003C>A), but neither were reported pathogenic so far.

## Discussion

ACC is a rare malignancy, with a high progression rate and a five-year survival rate less than 50% if locally advanced (6, 7). Most ACCs (approximately 60%) include functional hormone-secreting tumors, leading to tumor detection. Yet 40% of symptomatic patients with ACCs have CS and 24% have concurrent virilization (8). Very few of these tumors secrete primarily aldosterone, and less than 2.5% of ACCs exclusively produce aldosterone (9). Here, we describe a distinctive case of aldosterone-producing ACC. Her aldostern-renin rate(ARR) was 3.2. The European Society for Hypertension suggests a lower cutoff point (ARR between 1.12 and 2.7) (10). The plasma renin concentration(PRC) in this patient was not completely inhibited due to hyperthyroidism, which can increase renin levels (11). The underlying mechanisms may be that increased beta-adrenergic activity and catecholamine production contribute to the increase in mineralocorticoid function of the adrenal glands during thyrotoxicosis and upregulation of the renin angiotensin-aldosterone system (12).

Simultaneous occurrence of two different endocrine neoplasms is notably infrequent. Few cases have described concurrent ACCs and PTC. Only four cases matched closely with our patient among search results from the PubMed database (13–16). One case alone describes comorbidity of APAC and PTC (17). However, few of these patients underwent genetic testing. The comorbidity of both tumors appears suggestive of a hereditary component, with the most likely hereditary syndromes associated with ACC being MEN-1 (mutation in menin), LFS (TP53), and Beckwith-Wiedemann syndrome (11p15 and CDKN1C) (18). The known syndromes caused by a hereditary predisposition to multiple endocrine tumors do not specifically include the concurrence of PTC with ACC.

Genetic tests were performed to exclude known hereditary conditions that could potentially cause a combination of ACC and PTC. Analysis revealed heterozygous TP53 and MEN-1 mutations; however, neither were morbigenous so far. It remains uncertain whether the identified genes are causative for these two neoplasms; therefore, LFS and MEN-1 cannot be excluded.

GD is the most common cause of spontaneous thyrotoxicosis. Based on our investigation, there are no published cases of GD with ACC and PTC or with aldosterone-producing ACC. There is only one published case of a patient with synchronous GD and primary aldosteronism (19). Their perspective on the underlying mechanism associating the two diseases was unclear, but they suggested the possibility of autoimmune mechanisms, such as the actions of autoantibodies (19). GD and PTC may occur concomitantly. The incidence of PTC in GD has increased over time, and the rate of cancer associated with GD is higher than that in euthyroid controls (20–23). The etiology of the increased risk of PTC in GD is not fully understood; nonetheless, thyroid-stimulating immunoglobulin are thought to potentially contribute to this relationship (21, 22). The association between primary aldosteronism, PTC, and GD may be attributed to autoimmune disorders. However, the underlying mechanisms of aldosterone-producing ACCs and PTC with GD warrant further evaluation and research.

A previous meta-analysis revealed that GD has a significantly higher risk of associated multifocality/multicentricity and distant metastases at the time of cancer diagnosis but is not associated with PTC-related mortality or recurrence (24, 25). In our case, the PTC might also be multifocal since the patient had 3 similar solid nodules in the right thyroid lobe and one was confirmed PTC.

## Conclusion

There are few case reports documenting the comorbidity of aldosterone-producing ACC and PTC. When the concurrence of two different endocrine neoplasms occurs, it is imperatave for endocrinologists to consider the possibility of a underlying hereditary predisposition. Our genetic analysis found variants of the TP53 and MEN-1 gene; however, they have not yet been established as pathogenic. Based on our extensive review, there are no published cases of synchronized aldosterone-producing ACC, and PTC alongside with GD, and a link between these three diseases remains undetermined. Our case cannot eliminate the possibility of an as-yet-unidentified syndrome; more cases are needed to further elucidate this hypothesis.

## Data availability statement

The original contributions presented in the study are included in the article/supplementary material, further inquiries can be directed to the corresponding author.

## Ethics statement

The studies involving humans were approved by Hainan General Hospital Medical Ethics Committee. The studies were conducted in accordance with the local legislation and institutional requirements. The human samples used in this study were acquired from primarily isolated as part of your previous study for which ethical approval was obtained. Written informed consent for participation was not required from the participants or the participants’ legal guardians/next of kin in accordance with the national legislation and institutional requirements. Written informed consent was obtained from the individual(s) for the publication of any potentially identifiable images or data included in this article.

## Author contributions

YZ: Writing – original draft, Writing – review & editing, Conceptualization. JY: Writing – original draft, Writing – review & editing. CF: Data curation, Formal analysis, Writing – review & editing. FW: Formal analysis, Resources, Writing – review & editing. HL: Conceptualization, Supervision, Writing – review & editing. KC: Conceptualization, Supervision, Writing – review & editing.
